# Severe retinopathy of prematurity is associated with early post-natal low platelet count

**DOI:** 10.1038/s41598-020-79535-0

**Published:** 2021-01-13

**Authors:** Raffaele Parrozzani, Elisabetta Beatrice Nacci, Silvia Bini, Giulia Marchione, Sabrina Salvadori, Daniel Nardo, Edoardo Midena

**Affiliations:** 1 Department of Ophthalmology, University of Padova, Padova, Italy; 2 IRCCS-Fondazione Bietti, Rome, Italy; 3 Department of Woman’s and Child’s Health, University of Padova, Padova, Italy

**Keywords:** Retinopathy of prematurity, Inflammation

## Abstract

Pathophysiology of retinopathy of prematurity (ROP) still presents a gap. Lately blood tests parameters of premature infants have been measured at different times of ROP, attempting to detect correlations with ROP development and progression. So far, very early post-natal biomarkers, predictive of ROP outcome, have not been detected. Our purpose is to evaluate, in the earliest post birth blood sample, the correlation between routinely dosed blood parameters and ROP outcome. 563 preterm babies, screened according to ROP guidelines, were included and classified in conformity with ET-ROP study in “Group 1” (ROP needing treatment), “Group 2” (ROP spontaneously regressed) and “noROP” group (never developed ROP). The earliest (within an hour after delivery) blood test parameters routinely dosed in each preterm infant were collected. Platelet count was decreased in Group 1 versus noROP group (p = 0.0416) and in Group 2 versus noROP group (p = 0.1093). The difference of thrombocytopenic infants among groups was statistically significant (p = 0.0071). CRP was higher in noROP versus all ROPs (p = 0.0331). First post-natal blood sample revealed a significant thrombocytopenia in ROP needing treatment, suggesting a role of platelets in the pathophysiology and progression of ROP, possibly considering it as a predictive parameter of ROP evolution.

## Introduction

Retinopathy of prematurity (ROP) still represents one of the major causes of preventable childhood blindness worldwide^1,2^. It is a sight-threatening disease that can lead to bilateral retinal detachment when not timely and properly treated, with dreadful consequences. ROP manifestation has changed through the years, encompassing different “ROP generations”, with increased and reduced ROP incidence^3^. In high- and middle income countries, the improvement of neonatal care has led to a constant increase of the survival rate of extremely premature babies, giving rise to a new increase in ROP incidence, that may be hypothetically considered as a new ROP “epidemic”, with higher incidence of posterior and more aggressive forms, usually more prone to rapid worsening^4–6^. The pathophysiology of ROP has been widely investigated, and prematurity itself is the main, unavoidable risk factor for the development of ROP. Moreover, low gestational age (GA) and birth weight (BW) are the main drivers in the setting of prematurity^2^. Many other risk factors have been correlated to ROP worsening, such as persistent elevated oxygen saturation, bronchopulmonary dysplasia, sepsis, ventricular hemorrhage and genetic components^2,7,8^. Therefore, ROP can be defined as a multifactorial disorder, where prematurity does not represent the only explanatory driver. Indeed, in apparently equal premature newborns, different ROP outcomes may exist^8^, suggesting that some partially known genetic factors (such as those related to the Wnt transduction pathway) and other unknown factors may influence ROP course. The pathophysiology of ROP, even if widely investigated, remains partly unknown. One reason may be represented by the presence, in premature infants, of several concomitant systemic diseases, medications and interventions, such as surgical procedures, which may confound the correlation between risk factors and ROP. In the last years, some blood tests parameters of premature infants have been investigated to detect possible correlations with ROP development and progression^9–15^. These parameters were most often measured at different time point of ROP natural history, but very early post-natal biomarkers, possibly predictive of ROP outcome have not clearly been detected yet. Although systemic conditions have a great impact in the development of ROP, the identification of the earliest factors influencing ROP progression may be helpful for both ophthalmologists and neonatologists, to plan efficacious prevention and/or therapeutic strategies aiming to reduce the need of more invasive treatments for ROP^10^. The aim of this study was to evaluate in the earliest post birth blood sample, the correlation between routinely dosed blood parameters and retinopathy of prematurity (ROP) outcome in a preterm population.

## Methods

### Study population

This was a non-interventional, single center, retrospective study with a prospective enrolment, compliant with the tenets of the Declaration of Helsinki and approved by the local Institutional Review Board (Comitato Etico Azienda Ospedaliera di Padova, No. CESC2522P/2012). Informed consent was obtained from a parent or legal guardian of all infants. Preterm babies undergoing screening for ROP (gestational age (GA) ≤ 30 weeks and/or birth weight (BW) ≤ 1500 g) admitted to the Neonatal Intensive Care Unit (NICU) from August 2012 to March 2018 were consecutively included^16^. Clinical and demographic characteristics were collected, including GA and BW. Patients small for gestational age (SGA) (defined as babies < 10th centile for specific GA) were also recorded. Maximum ROP stage was classified according to International Classification of Retinopathy of Prematurity classification revisited^17^. The infants were also classified according to the ET-ROP study in Type 1 and Type 2 ROP^18^. For the purpose of this study, infants were then divided into three groups: “Group 1” included all infants affected by ROPs that required treatment, such as type 1 ROP (according to ET-ROP study classification) and aggressive posterior ROP (AP-ROP); “Group 2”, that included all infants who were classified as Type 2 ROP and mild ROPs (not satisfying Type 1 or Type 2 criteria according to ET-ROP study)^18^ and all underwent self-regression; finally “no ROP” group included infants who never developed any form of ROP.

The sub-division into three groups aimed to improve the comparison between the most severe ROPs, needing prompt treatment, and those who never developed any ROP, or never reached a severity requiring intervention.

### Blood parameters

The earliest (range few minutes to 1 h and thirty minutes after delivery) blood test parameters routinely dosed in each preterm infant were collected, including: platelet count (10^9^/L), white blood cell count (10^9^/L), white blood cells formula (including specific counts of neutrophils), lymphocytes and monocytes (10^9^/L) (also expressed in percentage of the whole blood count) and Reactive Protein C (CRP) (mg/L). The ratios between neutrophils/lymphocytes, lymphocytes/monocytes, and platelets count/lymphocytes were also obtained^19–22^. Thrombocytopenic infants (defined as having platelet count < 100 × 10^9^/l) were also recorded.

### Statistical analysis

Descriptive statistical methods were used for general parameters, such as GA, BW. All quantitative parameters were expressed as mean value ± standard deviation (SD), a range of minimum and maximum was also indicated; qualitative parameters were expressed as absolute and relative frequency in percentages. The comparison between infants who never developed any ROP (noROP group) and those who developed any ROP (Group 2 + Group 1) was performed by means of ANOVA test. The comparison was adjusted for GA, BW and SGA. When analyzing blood parameters, Group 1 was compared to Group 2 and noROP group, by means of ANOVA test and adjustment for GA, BW and SGA. When the comparison was statistically significant, multiple post-hoc Bonferroni tests among groups were performed. Blood parameters were also compared between Group 1 infants and all other infants (noROP + Group 2) by means of ANOVA test and adjustment for GA, BW and SGA. A relevant inter-dependence among blood parameters was present (Pearson’s correlation), except for monocytes, where a multivariate approach was used to evaluate the influence of blood parameters in the development of any ROP or Group 1 ROP. A stepwise logistic regression for selection of statistically significant results was used to evaluate the influence of specific parameters (risk or protective), net of their relationship with the other potentially correlated parameters. All statistical analyses were performed by means of SAS 9.3 software, on a personal computer (SAS Institute, Cary NC, USA). Statistically significance results were considered when p ≤ 0.05.

## Results

### Population characteristics

574 preterm infants were consecutively screened for inclusion. Of these, 11 (0.019%) patients were excluded at baseline for incomplete data at birth. Eventually, 563 preterm infants were included. Of these, 34 infants (0.06%) were characterized by data loss during follow-up (3 data loss in the Group 1 ROP (0.33%), 7 (0.045) in the Group 2 ROP, 24 (0.067) in the no-ROP group.) Data loss was caused by patient transfer to other centers with loss from follow-up (20 cases; 0.035%), hemolysis or other problems of the blood sample (4 cases; 0.07%) and death of the patient (10 cases; 0.017%).

**Figure 1:**
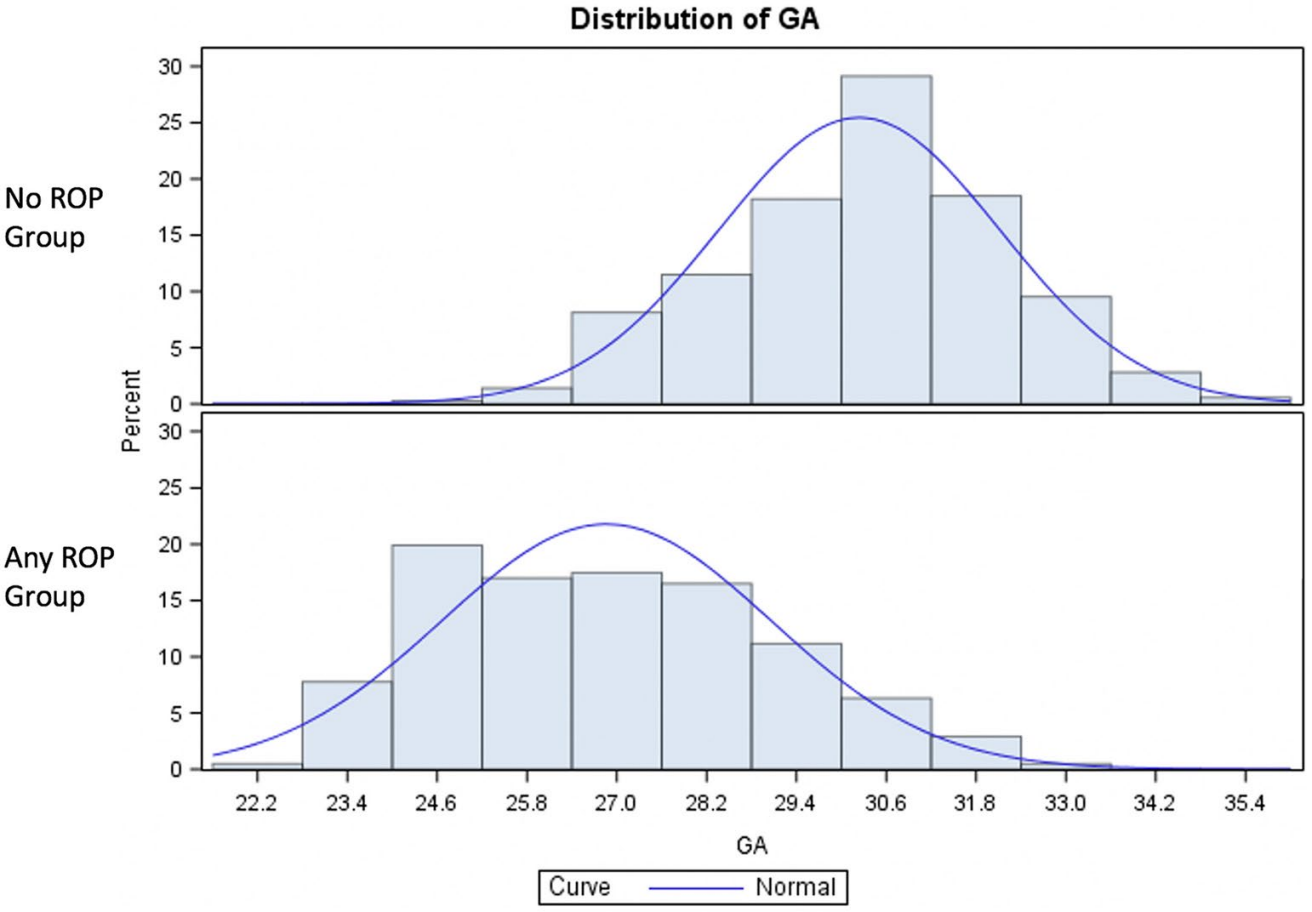
Distribution of Gestational Age in NoROP preterm babies and in all ROP group (Group 1 and Group 2). ANOVA test, p < 0.0001. *GA* gestational age.

**Figure 2:**
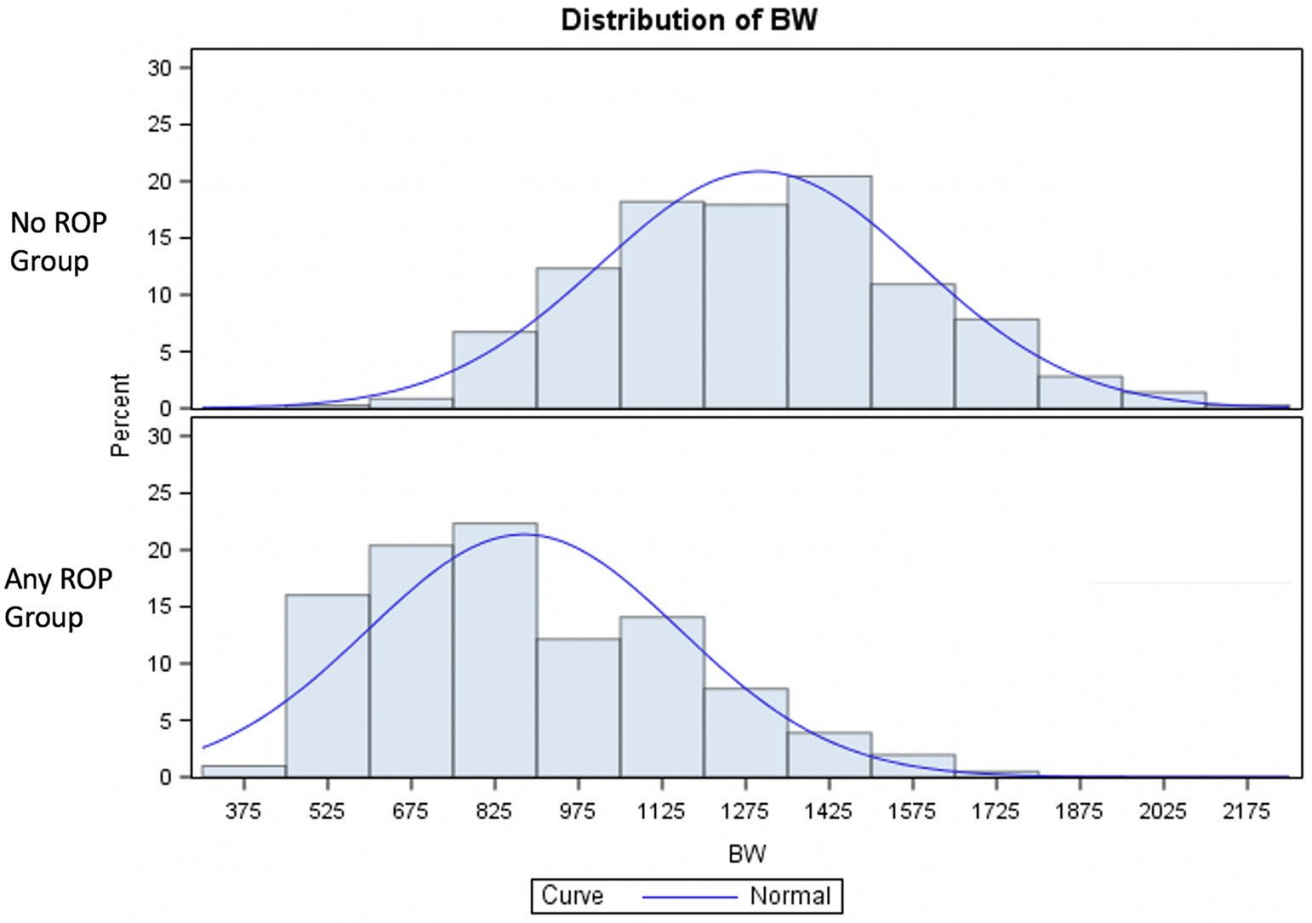
Distribution of birth weights in NoROP preterm babies and in all ROP group (Group 1 and Group 2). ANOVA test, p < 0.0001. Birth weights were adjusted for gestational age. *BW* birth weight.

Mean GA of the included infants was 28.72 ± 2.58 (range 22.68–35.57). Mean BW at birth was 1083.8 ± 329.62 (range 425.0–1985.0 g) (See details of the ROP groups in Table 1). 104 (18.47%) children were classified as SGA.196 (36.6%) infants developed any form of ROP, 52 developed severe ROP (Type 1 or AP-ROP: 9.2% of the total population and 25.2% of those who developed any ROP). All infants who developed Type 1 ROP or AP-ROP were treated. Of these, 35 patients (69%) received retinal laser photocoagulation, whereas 16 patients (31%) were treated by intravitreal anti-vascular endothelial grow factor injection followed by retinal laser photocoagulation at ROP recurrence, a mean of 46 ± 21 days after the intravitreal injection. Two infants developed retinal detachment (stage 4a) despite complete laser treatment and underwent vitreo-retinal surgery. No child developed stages 4b or 5. The infants who developed any ROP were significantly smaller for GA and BW than no ROP babies (p < 0.0001 for both) (Figs. 1 and 2), and infants of Group 1 ROP were significantly smaller for GA and BW than Group 2 ROP babies (p < 0.0001 and p = 0.0461, respectively, Table 2). An increased incidence of SGA infants was documented in the any ROP group compared to the no ROP group (p = 0.0249) and in Group 1 ROP compared to no ROP group (p = 0.0076) (Table 2).

**Table 1:** Comparison of mean birth weight, gestational age and blood parameters measured in infants without and with retinopathy of prematurity.

ROP	Parameter	Mean value	Std Dev	Minimum	Median	Maximum	P-value*
No ROP (n = 357)	BW	1299.532	286.743	425	1290	1985	
	GA	30.238	1.1881	24.429	30.286	35.571	
	CRP	5514	10.624	0	2.9	112	
	WBC	9315	8807	1.59	7.91	130	
	NEU	5055	6651	0.28	3715	91	
	LYM	3086	1507	0.31	2.73	9.26	
	MONO	1399	6796	0.03	0.855	121	
	PLT	210.157	72.371	1.37	210	407	
	NEU/LYM	1915	2084	0.133	1314	19.774	
	LYM/MONO	5469	944	0.053	3353	118.333	
	PLT/LYM	83.629	51887	0.333	73.75	384.127	
Any ROP (n = 206)	BW	876.772	280.033	425	815	1735	< 0.0001
	GA	26.861	2198	22.714	27	32.571	< 0.0001
	CRP	9198	18.465	0	2.9	219.8	0.0331
	WBC	12.366	14.923	1.45	7.94	137.22	0.6336
	NEU	7455	10.669	0.43	3.89	91.94	0.445
	LYM	2815	1847	0.15	2.34	11.61	0.4454
	MONO	1315	1585	0.03	0.955	12.75	0.5563
	PLT	190.829	79.408	1.48	182	442	0.0209
	NEU/LYM	3059	4141	0.163	1655	35.577	0.6669
	LYM/MONO	5133	12.482	0.401	2512	119.429	0.802
	PLT/LYM	89.842	60.828	0.718	78.385	393.333	0.9603

*Std Dev* standard deviation, *ROP* retinopathy of prematurity, *BW* birth weight, *GA* gestational age, *CRP* C-reactive protein, *WBC* white blood cell count, *NEU* neutrophils, *LYM* lymphocytes, *MONO* monocytes, *PLT* platelets.

*ANOVA test adjusted for gestational age, birth weight and small for gestational age.

**Table 2:** Comparison of clinical parameters (GA, BW, SGA, CRP and PLT) among the three groups NoROP, Group 2 and Group 1.

	No ROP	Group 2	Group 1	p-value*		
				No ROP vs G1	No ROP vs G2	G1 vs G2
GA (weeks)	30.238 ± 1.881	27.357 ± 2.175	25.390 ± 1.506	< 0.0001	< 0.0001	< 0.0001
BW (grams)	1299.53 ± 286.74	939.56 ± 272.66	690.80 ± 212.12	< 0.0001	< 0.0001	0.0461
SGA (Number; %)	56 (15.68%)	32 (16.32%)	16 (30.78%)	0.0076	0.8439	0.0191
CRP (mg/L)	5.514 ± 10.624	8.922 ± 12.028	9.998 ± 13.058	0.0919	0.1539	0.1199
PLT (10^9^/L)	210.157 ± 72.371	193.813 ± 76.773	181.878 ± 87.063	0.0416	0.1093	0.8078

*ROP* retinopathy of prematurity; *BW* birth weight; *GA* gestational age; *SGA* small for gestational age; *CRP* C-reactive protein; *PLT* platelets; *G1* Group 1 including Type 1 ROP and Aggressive Posterior ROP, requiring treatment; *G2* Group 2 including Type 2 ROPs and mild ROPs with self regression.

### Blood test parameters

**Figure 3:**
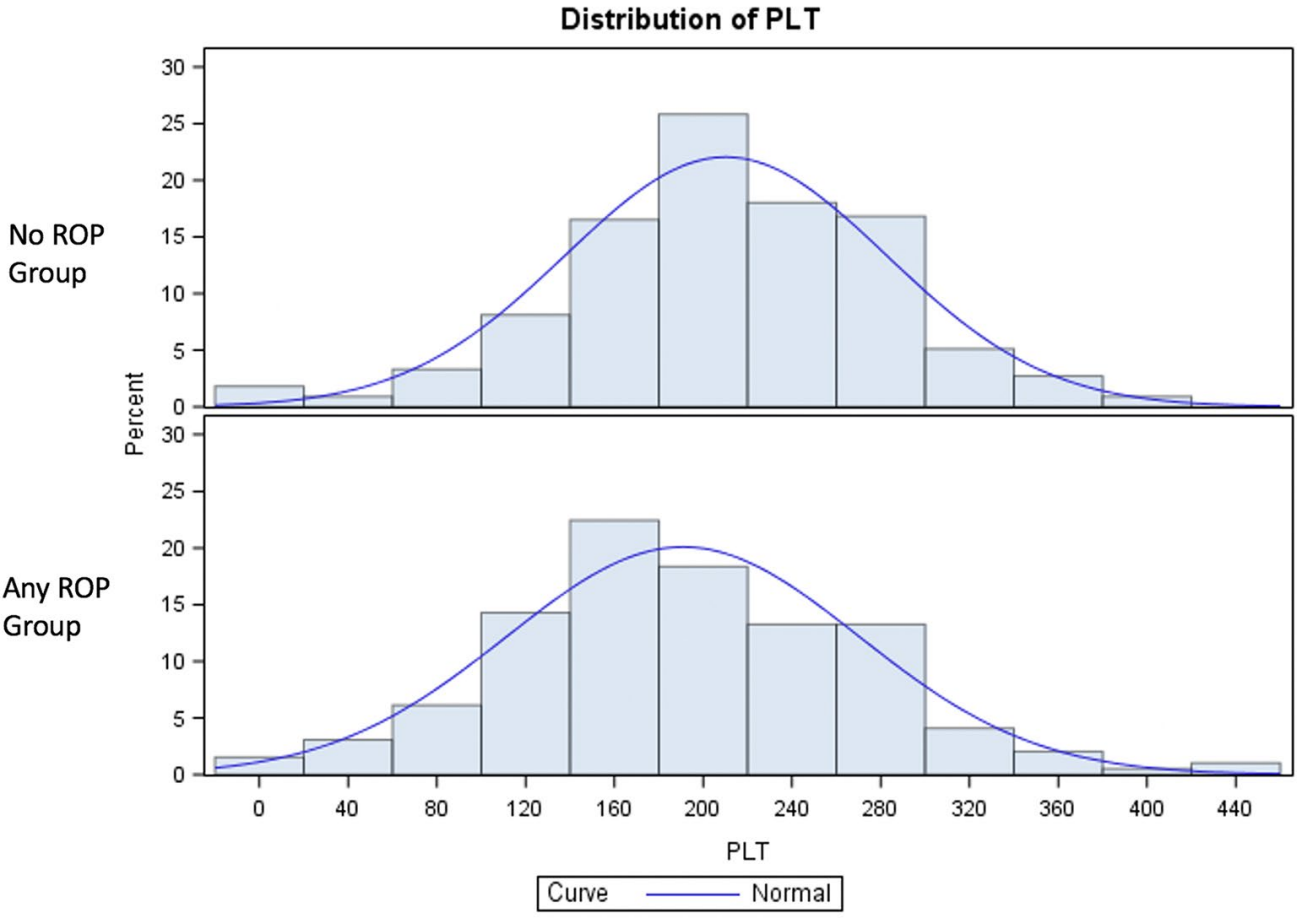
Distribution of platelet count (10^9^/L) in NoROP group and in infants who developed any form of ROP. ANOVA test, p = 0.0209. Platelet count was adjusted for BW and GA. *PLT* platelets, *BW* birth weight, *GA* gestational age.

Mean values and standard deviation of the collected blood parameters are reported in Tables 1 and 2. Among the measured earliest post birth blood test parameters, CRP and platelet count showed statistically significant differences correlated to ROP presence (p < 0.05). Specifically, mean value of CRP showed a statistically significant difference when comparing all ROP patients (Group 1 + Group 2) to noROP group (ANOVA test, adjusted for GA, BW and SGA, p = 0.0331), with the highest mean value in Group 1. A trend of increase of CRP value from noROP to Group2 and Group1 was observed (5.5, 8.9, 9.9 mg/L respectively), without statistically significant difference when comparing each group separately (p > 0.05) (Table 2). Baseline platelets count showed a significant difference between noROP group and all ROPs (Group 1 + Group 2) (p = 0.0209, Table 1, Fig. 3). Platelets count was significantly reduced in Group1 versus noROP (p = 0.0416), and a progressive reduction trend was found in the three groups (Table 2). Forty patients (6.9%) were classified as thrombocytopenic (platelet count < 100 × 10^9^/l); 19 of 333 infants of the noROP group (5.71%), 12 of 147 of the Group 2 (8.16%), and 9 of 49 of the Group 1 group (18.37%) (see Table 3). The difference of thrombocytopenic infants among groups was statistically significant (Chi-square test, p = 0.0071). All other measured parameters, including the ratio of white blood cells count, did not show any statistically significant difference among groups (p > 0.05).

**Table 3:** Number of cases of thrombocytopenia divided in the three groups: NoROP, Group 1 and Group 2.

	Thrombocytopenia^a^	
	Yes	No
NoROP, n/tot (%)	19/333^b^ (5.71%)	314/333 (94.29%)
Group 2 ROP, n/tot (%)	12/147^c^ (8.16%)	135/147 (91.84%)
Group 1 ROP, n/tot (%)	9/49^d^ (18.37%)	40/49 (81.63%)

*NoROP* premature infants who never developed any form of ROP, *Group 2* Type 2 ROP and mild ROPs with self regression of the disease, *Group 1* Type 1 ROP and Aggressive posterior ROP that needed treatment,* ROP* retinopathy of prematurity.

Chi-square test, p = 0.0071.

^a^34 data loss.

^b^3 data loss in the Group 1 ROP.

^c^7 data loss in the Group 2 ROP.

^d^24 data loss in the no-ROP group.

## Discussion

The mechanism leading to ROP development is mainly attributed to prematurity itself and oxygen delivery. Despite the pathophysiology of ROP has been widely investigated it still remains partly unknown. There are apparently similar premature babies, sharing similar GA and BW, who, however, have a completely different ROP course, with some cases needing treatment, and others that regress spontaneously. This opposite evolution of ROP remains unexplained. However, several correlations have been found between Type 1 ROP development and conditions such as sepsis^14^, but the mediators and the exact pathologic mechanisms driving the retina to develop a severe form of ROP are not fully understood. Human models to detect possible biomarkers of ROP progression, are tricky to be obtained, due to the extreme fragility and comorbidities of these type of infants. Therefore, most studies have been conducted on animal models, using oxygen-induced retinopathy^23,24^. However, in the last years, the need to improve the management of this potentially blinding disease has led several Authors to investigate in vivo the possible correlations between routinely tested blood parameters and ROPoutcome^11–15^. Specifically, these authors have shown that a reduced platelet count and thrombocytopenia (platelets < 100 × 10^9^/l or < 15010^9^/l) was correlated to severe ROP development^11–15^, suggesting a possible role of platelets in the pathogenesis and clinical course of ROP. The correlations of these systemic biomarkers with severe ROP were obtained at different time points of ROP natural history. This approach may prevent from detecting an early predictive serum biomarker. Moreover, blood parameters are influenced by general conditions, systemic treatment, interventions, that may start or change during the first days or months of life of these infants. Therefore, our study analyzed the earliest blood test parameters in order to avoid these confounding factors. Moreover, our population was higher in number compared to other studies^11–13^. Our data demonstrated that premature infants developing severe ROP needing treatment are significantly smaller for GA and BW than noROP infants, confirming the importance of these parameters as main drivers of ROP. When looking at blood test results, the earliest available platelet count (performed right after delivery) showed statistically significant differences among groups. It was significantly reduced in those infants who later developed severe ROP requiring treatment, compared to those who never developed any ROP. This result, in accordance with previous studies^11–15,25,26^, suggests that early platelet count, among the routinely performed blood parameters, may help to detect, far in advance, those infants who may be more prone to ROP worsening. More specifically, our study demonstrated that there was a trend of reduction of platelet count from noROP group to Group 2 and Group 1 ROP. However, the role of platelets in ROP is not completely understood. As in the whole human body, they might play different—or even opposite—functions in the peripheral tissues: pro-angiogenic or anti-angiogenic. This is due to the presence of several granules inside platelets, containing different molecules, with pro-angiogenic function, such as vascular endothelial growth factor (VEGF) and angiogenesis inhibitor molecules, such as endostatin, that are supposed to be stored in different alpha granules and released alternatively^27,28^. The separate packaging of angiogenesis regulators into morphologically distinct populations of α-granules in platelets may provide a mechanism by which platelets can locally stimulate or inhibit angiogenesis. Our first hypothesis is that in the retinal tissue, during vascularization phases, platelets may play a “scavenger” role in the perfused retina, by removing VEGF^27,28^. This would explain why a reduced number of platelets may be correlated to severe ROP development, as the scavenger role is missed in low platelet count, especially in thrombocytopenia. A recent work showed that any episode of thrombocytopenia by the time of 30 or more weeks of postmenstrual age was independently associated with severe ROP requiring treatment^11^. These Authors also showed that low platelets count during neovascularization phase of ROP (also known as second phase) is significantly associated to severe ROP development^11^. Platelets showed an anti-angiogenic effect on retinal endothelial cells that reduced VEGF-A production. However, this was an animal model (mouse oxygen-induced retinopathy), and these data have been just partially confirmed in human studies were many other factors may be involved in the same subject^11^.

Although the hypothesis that platelets may play a scavenger role in the perfused retina by removing VEGF during phase 2 ROP (the period of neovascularization)^11^, in our study platelets count was determined at birth, the phase 1 ROP. In the preterm neonate a large part of the retina is still avascular in this first phase, needing to be further vascularized. Therefore, less suppression of VEGF as a result of low platelets count would then be theoretically an advantage. A second possibility may be that at birth (phase 1 ROP) platelets may have a more stimulating function in angiogenesis by delivering IGF-1, and that low platelets may cause an imbalance between regulation mediators^29,30^. This second hypothesis fits well with the timing of sampling in our study (at birth), as well with the hypothesis that this parameter may represent a risk factor of severe ROP independently from other systemic conditions that infants may encounter during the first months of life.

In our study also CRP showed a significant difference between noROP group and the other ROP groups (Group 1 and Group 2 ROP). Despite an increasing trend from noROP to Group 2 ROP and Group 1 ROP group, the statistically significance was lost when comparing any single group separately. This may be due to the low specificity of CRP value, which may be influenced by several systemic conditions. However, this data seems to confirm a previous report on the role of inflammation as a negative influencer of ROP progression^14^. Possible limitations of this study may be represented by the lack of a longitudinal analysis of blood tests results to monitor possible changes in relation to ROP evolution, and by the absence of correlation with systemic conditions that could also have influenced the development of ROP (early or late onset sepsis, necrotizing enterocolitis, prolonged mechanical ventilation). A prospective study taking into account these confounding factors is under way. However, the aim of this study was to find an early parameter that could represent an independent risk factor of severe ROP and may be predictive of ROP outcome (as early as the time of delivery itself), when not yet influenced by pharmacological treatments, surgical interventions, or further systemic conditions. Nevertheless, also in presence of a possible predictive parameter allowing a precocious identification of “at risk” babies, infants with low risk at birth may encounter many other severe conditions that can still increase their risk to develop severe ROP.

The correlation we found between low platelet count and the development of severe ROP agrees with previous results, and seems to suggest that a simple blood test, just after birth, may be predictive of an unfavorable course of ROP and may help to elucidate the pathophysiology of ROP. This would ideally improve the management of this potentially blinding condition. The precocious identification of “at risk” babies by means of a simple blood test, would also help to detect, far in advance, those infants that could more easily develop a severe form of ROP, and must be monitored with more attention. Moreover, if the role of platelets would be confirmed in ROP pathogenesis, this could lead to new hypothesis in ROP prevention and treatment strategies that could be of interest also for neonatologists.

## References

[1] Solebo AL, Teoh L, Rahi J (2017). Epidemiology of blindness in children. Arch. Dis. Child..

[2] Kim SJ (2018). Retinopathy of prematurity: A review of risk factors and their clinical significance. Surv. Ophthalmol..

[3] Smith BT, Tasman WS (2005). Retinopathy of prematurity: Late complications in the baby boomer generation (1946–1964). Trans. Am. Ophthalmol. Soc..

[4] Quinn GE, Gilbert C, Darlow BA, Zin A (2010). Retinopathy of prematurity: An epidemic in the making. Chin. Med. J. (Engl)..

[5] Adams GG (2017). Treatment trends for retinopathy of prematurity in the UK: Active surveillance study of infants at risk. BMJ Open..

[6] Gilbert C (2019). Epidemiology of ROP update—Africa is the new frontier. Semin. Perinatol..

[7] Chang JW (2019). Risk factor analysis for the development and progression of retinopathy of prematurity. PLoS ONE.

[8] Swan R (2018). The genetics of retinopathy of prematurity: A model for neovascular retinal disease. Ophthalmol. Retina..

[9] Poryo M (2019). Nucleated red blood cells and serum lactate values on days 2 and 5 are associated with mortality and morbidity in VLBW infants. Wien Med. Wochenschr..

[10] Sola-Visner M, Bercovitz RS (2016). Neonatal platelet transfusions and future areas of research. Transfus. Med. Rev..

[11] Cakir B (2018). Thrombocytopenia is associated with severe retinopathy of prematurity. JCI Insight..

[12] Jensen AK, Ying GS, Huang J, Quinn GE, Binenbaum G (2018). Longitudinal study of the association between thrombocytopenia and retinopathy of prematurity. J. AAPOS..

[13] Sancak S, Toptan HH, Yildirim TG, Karatekin G, Ovali F (2019). Thrombocytopenia as a risk factor for retinopathy of prematurity. Retina..

[14] Lundgren P (2017). Aggressive posterior retinopathy of prematurity is associated with multiple infectious episodes and thrombocytopenia. Neonatology..

[15] Lundgren P (2019). Erythropoietin serum levels, versus anaemia as risk factors for severe retinopathy of prematurity. Pediatr. Res..

[16] Fierson WM (2018). Screening examination of premature infants for retinopathy of prematurity. Pediatrics.

[17] International Committee for the Classification of Retinopathy of Prematurity (2005). The international classification of retinopathy of prematurity revisited. Arch. Ophthalmol..

[18] Early Treatment For Retinopathy Of Prematurity Cooperative Group (2003). Revised indications for the treatment of retinopathy of prematurity: Results of the early treatment for retinopathy of prematurity randomized trial. Arch. Ophthalmol..

[19] Yang YC, Mao J (2018). Value of platelet count in the early diagnosis of nosocomial invasive fungal infections in premature infants. Platelets.

[20] Kurtul BE (2015). Serum neutrophil-to-lymphocyte ratio in retinopathy of prematurity. J. AAPOS..

[21] Jiang Y, Zang M, Li S (2017). Serum PLR and LMR in Behcet's disease. Can they show the disease activity?. Medicine (Baltimore)..

[22] Zhang H (2018). Association of systemic inflammation score with atrial fibrillation: A case-control study with propensity score matching. Heart Lung Circ..

[23] Li N, Gao S, Wang J, Zhu Y, Shen X (2019). Anti-apoptotic effect of interleukin-17 in a mouse model of oxygen-induced retinopathy. Exp. Eye Res..

[24] Singh C (2019). Serine and 1-carbon metabolism are required for HIF-mediated protection against retinopathy of prematurity. JCI Insight..

[25] Vinekar A (2010). Do platelets have a role in the pathogenesis of aggressive posterior retinopathy of prematurity?. Retina..

[26] Jensen AK, Ying GS, Huang J, Quinn GE, Binenbaum G (2011). Thrombocytopenia and retinopathy of prematurity. J. AAPOS..

[27] Italiano JE (2008). Angiogenesis is regulated by a novel mechanism: Pro- and antiangiogenic proteins are organized into separate platelet alpha granules and differentially released. Blood.

[28] Folkman J (2007). Angiogenesis: An organizing principle for drug discovery?. Nat. Rev. Drug Discov..

[29] Christensen RD (2015). Thrombocytopenia in small-for-gestational-age infants. Pediatrics.

[30] Razak A, Faden M (2019). Association of small for gestational age with retinopathy of prematurity: A systematic review and meta-analysis. Arch. Dis. Child Fetal Neonatal..

